# Case Report: A long-term response of immunotherapy combined with anti-angiogenesis therapy in a patient with dMMR metastatic colorectal cancer after ICI failure

**DOI:** 10.3389/fonc.2025.1553380

**Published:** 2025-04-09

**Authors:** Qianwen Huang, Xiaoling Zheng, Wenshen Xu

**Affiliations:** ^1^ Department of Medical Oncology, Boluo County People’s Hospital, Huizhou, China; ^2^ Department of Pharmacy, Boluo County People’s Hospital, Huizhou, China

**Keywords:** metastatic colorectal cancer, high microsatellite instability, deficient mismatch repair, immune checkpoint inhibitors, anti-angiogenesis therapy

## Abstract

**Background:**

Immune checkpoint inhibitors (ICIs) have demonstrated significant efficacy in patients with metastatic colorectal cancer (mCRC) characterized by high microsatellite instability (MSI-H) or deficient mismatch repair (dMMR). However, most patients experience intrinsic or acquired resistance. The need for treatment for patients with MSI-H/dMMR mCRC remains unmet. Here, we report the case of a patient with dMMR mCRC who achieved a durable therapeutic benefit from the combination of ICI and angiogenesis inhibitor after ICI failure.

**Case presentation:**

A 40-year-old Chinese woman diagnosed with cT4N2M1b mCRC characterized by dMMR attributed to MLH-1 and PMS-2 deficiency, along with KRAS mutation. Primarily, the patient was treated with a combination of Chinese medicine and XELOX and underwent disease progression. Due to dMMR status, this patient then received single-agent camrelizumab. Unfortunately, disease progression was observed after two cycles of treatment. Subsequently, she received camrelizumab combined with bevacizumab. After treatment, the patient achieved a complete response, and the disease was sustainably controlled with a progression-free survival (PFS) of 3 years and counting.

**Conclusions:**

This report demonstrates that the combination of ICI and anti-angiogenesis therapy can induce a powerful and durable antitumor response in patients with ICI-resistant MSI-H/dMMR mCRC, which is worthy of further research.

## Introduction

Colorectal cancer (CRC) is ranked among the third most prevalent cancers and is the second leading cause of cancer-related mortality (1). It has been highly associated with genetic factors, non-cancerous diseases including colorectal polyps and adenomas, and lifestyle factors such as high consumption of animal protein and fat. Previous researchers identified that approximately 25% of the patients diagnosed with CRC exhibit the occurrence of metastatic CRC (mCRC) at the initial stage of diagnosis, and 50% of the patients progress to metastases (2). Cancers characterized by high microsatellite instability (MSI-H) or deficient mismatch repair (dMMR) define a molecular subgroup of CRC accounting for approximately 15% of all CRC and 5% of mCRC patients (3, 4). The emergence of immune checkpoint inhibitors (ICIs) has revolutionized the therapeutic perspective and prognosis of MSI-H/dMMR mCRC (5–7). Numerous studies have demonstrated the advantages of anti-programmed death-1 (PD-1) when used as a single-agent therapy or in combination with anti-cytotoxic T lymphocyte antigen-4 (CTLA-4) in the treatment of MSI-H/dMMR mCRC (5, 8). Therefore, the single-agent and dual ICIs have been effectively integrated into MSI-H/dMMR mCRC treatment.

Although the ICIs serve substantial potential in MSI-H/dMMR mCRC patients, approximately 30%–40% of the patients do not respond to ICI treatment (5). In some patients, disease progression may occur after the initial response (9). Therefore, the need for treatment for the patient with MSI-H/dMMR mCRC remains inadequately addressed.

Currently, researchers are focusing on exploring the potential advantages of integrating immunotherapy with other therapies including targeted therapy and chemotherapy, to overcome ICI resistance or induce a stronger anti-tumor immune response in MSI-H/dMMR mCRC. Multiple preclinical studies revealed that anti-angiogenesis therapy may transform the immunosuppressive microenvironment of tumors into an immune-supportive microenvironment. Combining this therapy with immunotherapy can induce a synergistic effect and develop enduring antitumor immunity (10–12). This report elaborates on a case of a patient with dMMR mCRC, exhibiting prolonged response to the integration of camrelizumab and bevacizumab following the progression from the first-line chemotherapy and the second-line single-agent immunotherapy.

## Case description

A 40-year-old Chinese woman was diagnosed with cT4N2M1b mCRC located in the sigmoid colon. A colonoscopy assessment was conducted on a patient, and a stenosing mass at the sigmoid colon was identified. A biopsy, performed on 3 March 2021, confirmed the presence of moderately differentiated adenocarcinoma. A computed tomography (CT) scan of the abdomen was performed, and a stenosing neoplasm at the sigmoid colon, a large accumulation of intestinal contents, uterine infiltration, and multiple metastatic tumors in the liver were observed. The patient experienced bloating, abdominal pain, and the absence of bowel movements. Following the diagnosis of intestinal obstruction, the patient underwent a palliative transverse colostomy on March 9, 2021. The patient was initially administered the combination of Chinese medicine and XELOX [Oxaliplatin (L-OHP) 130 mg/m^2^ ivgtt d1 and capecitabine 1,000 mg/m^2^, bid, po, d1-14]. This treatment was conducted every 3 weeks from 30 March 2021 to 19 August 2021, occurring in six cycles.

**Figure 1:**
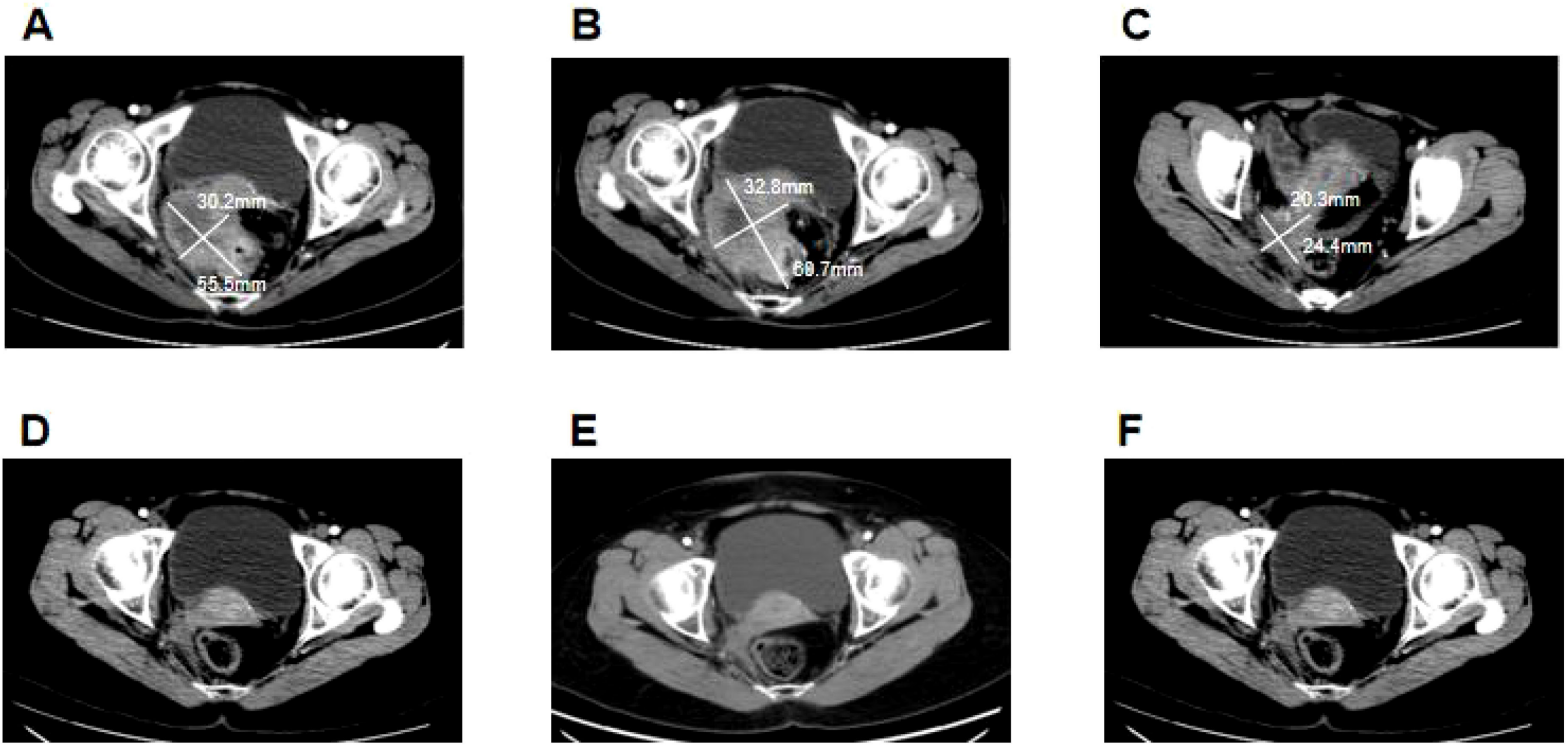
Clinical responses to treatment. **(A)** Before camrelizumab treatment, the size of the colon tumor was about 5.6 cm × 3 cm. **(B)** After two cycles of camrelizumab treatment, the size of the colon tumor increased to approximately 6.1 cm × 3.3 cm. **(C)** After six cycles of camrelizumab treatment, in combination with bevacizumab, the size of the colon tumors decreased significantly to about 2.4 cm × 2 cm. **(D)** After being treated with camrelizumab and bevacizumab for 1 year, the colon tumor reduced progressively, exhibiting a complete response. **(E)** After 2 years of treatment with camrelizumab and bevacizumab, the colon lesions of the patient maintained a complete response. **(F)** The patient maintained a complete response after stopping the 1-year treatment.

**Figure 2:**
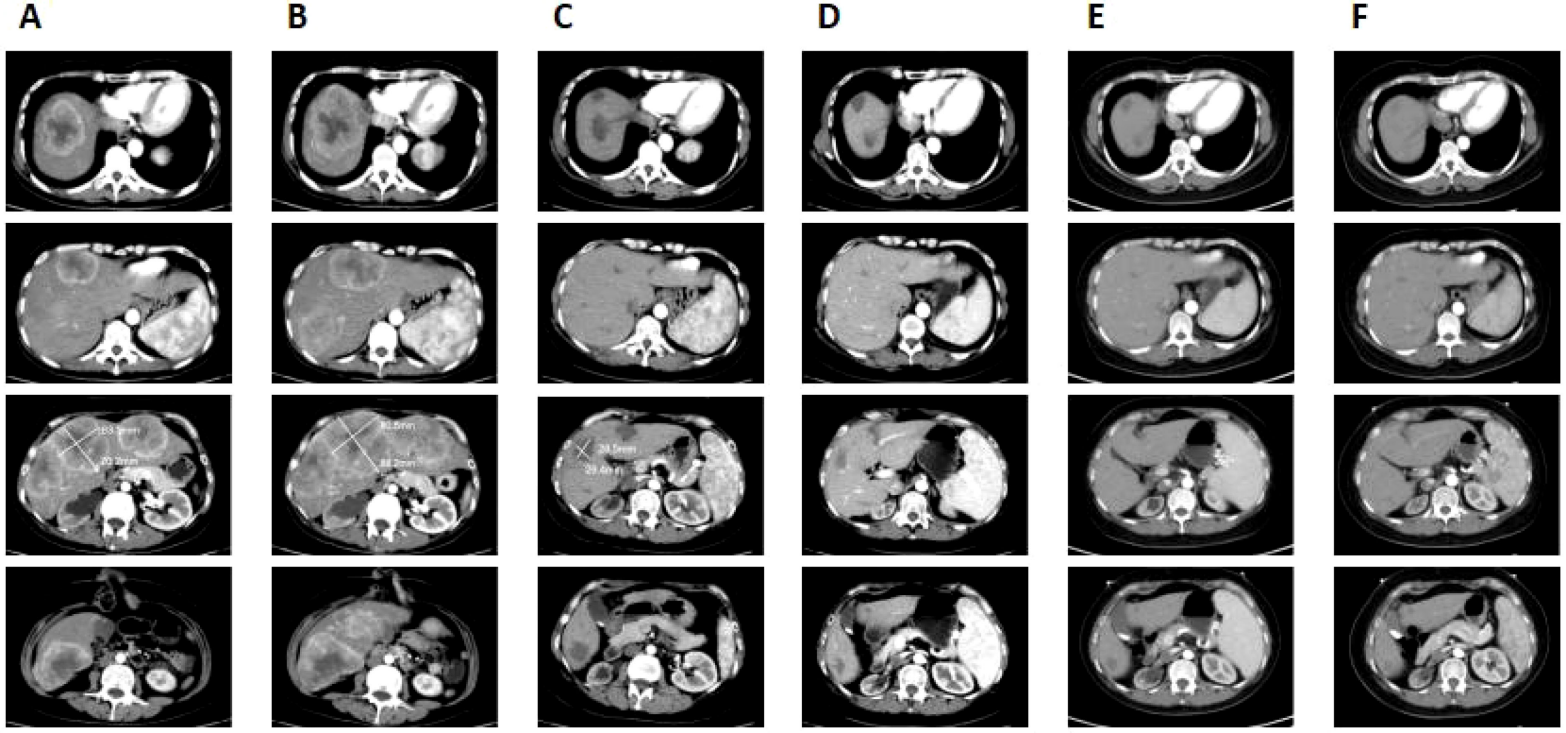
Clinical responses to the treatment. **(A)** Before camrelizumab treatment, multiple metastatic tumors were seen in the liver and the largest lesion was about 7 cm × 6.4 cm. **(B)** After two cycles of camrelizumab treatment, the liver metastases increased in size and number, with the largest lesion measuring 8.8 cm × 8.1 cm. **(C)** After the six cycles of camrelizumab treatment combined with bevacizumab, the size of liver metastases was reduced, with the largest one measuring 2.9 cm × 2.6 cm and some lesions had disappeared. **(D)** Following treatment with camrelizumab and bevacizumab for 1 year, the size of the lesion in the liver was progressively decreased, with no apparent tumor activity, achieving a complete response. **(E)** After 2 years of camrelizumab and bevacizumab treatment, the patient maintained a complete response. **(F)** The lesion in the liver remained unchanged after stopping the treatment for 1 year.

On 25 September 2021, the patient came to our hospital and presented a Karnofsky performance score (KPS) of 60, indicating that she was unable to work and needed help with self-care activities. She had suffered from abdominal pain and fatigue for 2 weeks. The enhanced CT scan revealed a thickening on the intestinal wall of the sigmoid colon accompanied by a mass formation (**Figure 1A**), and the tumor size was about 5.6 cm × 3 cm. This condition invaded the uterus, bladder, and lower right ureter and caused hydronephrosis. Additionally, large, multiple metastatic tumors were also observed in the liver (**Figure 2A**), and the largest lesion was about 7 cm × 6.4 cm. Her blood tests indicated severe anemia with hemoglobin (Hb) of 46 g/L and a normal hepatorenal function. The levels of tumor markers were recorded as 430.6 ng/ml, 704.9 U/ml, and >300 U/ml for carcinoembryonic antigen (CEA), carbohydrate antigen 19-9 (CA19-9), and carbohydrate antigen 72-4 (CA72-4), respectively (**Table 1**). We performed an immunohistochemical (IHC) stain and genetic testing on the tissue obtained from the previous biopsy, revealing a dMMR (MLH-1 and PMS-2 deficient) and KRAS mutation.

**Table 1:** The changes in tumor marker levels.

Date	CEA	CA19-9	CA72-4
September 25, 2021	430.6 ng/ml	704.9 U/ml	> 300 U/ml
November 18, 2021	804.3 ng/ml	> 1000 U/ml	> 300 U/ml
January 6, 2022	57.81 ng/ml	199.6 U/ml	35.32 U/ml
April 6, 2022	1.74 ng/ml	11.95 U/ml	0.956 U/ml
December 10, 2022	1.65 ng/ml	12.61 U/ml	1.04 U/ml
December 10, 2023	1.75 ng/ml	10.93 U/ml	0.684 U/ml
November 14, 2024	2.33 ng/ml	10.98 U/ml	1.21 U/ml

CEA, carcinoembryonic antigen; CA19-9, carbohydrate antigen 19-9; CA72-4, carbohydrate antigen 72-4. The normal healthy ranges of the markers: CEA, 0–5 ng/ml; CA19-9, 0–39 U/ml; CA72-4, 0–6.9 U/ml.

The patient was treated for severe anemia by a blood transfusion and was administered opioid analgesics to manage abdominal pain. The condition of the patient was confirmed as a progressive disease (PD). Based on the dMMR tumors, PD-1 blockade camrelizumab (200 mg, every 3 weeks) was initiated on 6 October 2021. After two cycles of treatment, an enhanced CT scan conducted on 18 November 2021 showed that the colon tumor had grown from 5.6 cm × 3 cm to 6.1 cm × 3.3 cm (**Figure 1B**), and the size and number of liver metastases increased (**Figure 2B**), and the largest lesion grew from 7 cm × 6.4 cm to 8.8 cm × 8.1 cm. The tumor markers were CEA: 804.3 ng/ml, CA19-9: >1,000 U/ml, and CA72-4: >300 U/ml (**Table 1**), denoting a significant increase compared to the initial records. Moreover, the patient reported that abdominal pain and fatigue were more pronounced. Upon completion of the two cycles of camrelizumab treatment, the response results indicated PD.

Considering the bad condition of the patient and economic constraints, chemotherapy was not an option, so the patient received camrelizumab (200 mg) in combination with angiogenesis inhibitor bevacizumab (400 mg) every 3 weeks on 23 November 2021. Following the two cycles of treatment, the levels of CEA, CA19-9, and CA72-4 had dropped to 57.81 ng/ml, 199.6 U/ml, and 35.32 U/ml, respectively (**Table 1**). The symptoms of abdominal pain and fatigue in the patient significantly improved and the patient continued with the four cycles of camrelizumab and bevacizumab treatment. On 6 April 2022, an enhanced CT revealed a partial response (PR) exhibiting that the sizes of the colon tumor were reduced from 6.1 cm × 3.3 cm to 2.4 cm × 2 cm (**Figure 1C**) and some liver lesions became invisible, the largest lesion was reduced from 8.8 cm × 8.1 cm to 2.9 cm × 2.6 cm (**Figure 2C**). CEA, CA19-9, and CA72-4 levels decreased to normal values (**Table 1**), abdominal pain improved and drugs were no longer required. Ultimately, the patient exhibited an improved quality of life, with a KPS of 90.

Subsequently, the patient was consistently administered camrelizumab and bevacizumab treatment. An enhanced CT scan conducted on 10 December 2022, revealed a constant reduction in the size of the colon tumor (**Figure 1D**) and a consistent reduction in liver metastases (**Figure 2D**). The levels of CEA, CA19-9, and CA72-4 remained within normal range (**Table 1**). The residual lesions were considered to have no tumor activity. According to the modified response evaluation criteria on solid tumors (mRECIST), the patient’s disease was confirmed to have achieved a complete response (CR). Therefore, we recommended conducting a biopsy on liver lesions to evaluate the activity of the tumor but the patient declined this offer.

On 10 December 2023, the patient had been on the combined treatment for 2 years and an assessment with an enhanced CT scan showed a sustained stable colon tumor (**Figure 1E**) and liver metastases (**Figure 2E**). The levels of tumor markers were within normal ranges (**Table 1**). Afterwards, the patient stopped taking the treatment and she was followed up. On 19 November 2024, a follow-up enhanced CT revealed that the size of the colon lesion (**Figure 1F**) and liver metastases (**Figure 2F**) remained relatively unchanged. The levels of tumor markers were effectively controlled (**Table 1**), and no adverse events related to immunotherapy and antiangiogenesis therapy were observed. This patient maintained a CR until the submission of this manuscript, achieving a progression-free survival (PFS) of 3 years and counting.

## Discussion

We presented a case demonstrating the clinical efficacy of camrelizumab in combination with bevacizumab in a patient with dMMR mCRC who experienced disease progression after the first-line chemotherapy and second-line single-agent ICI treatment. The patient achieved CR after 12 months of treatment and maintained this status up to the submission of this manuscript, with a PFS of 3 years and counting.

Although ICIs demonstrate significant clinical efficacy in MSI-H/dMMR mCRC, the overall response rates (ORR) of single-agent and dual ICI treatment are 44% and 69%, respectively. In addition, approximately >50% of the patients experienced intrinsic or acquired resistance (5, 6, 9).

How to overcome resistance to ICIs and maximize the treatment potential of ICIs is a major concern in clinical research. Combination therapy is a promising treatment strategy. In recent years, researchers have been concentrating on the application of immunotherapy in combination with other treatment strategies, including targeted therapy and chemotherapy, which result in therapeutic synergy (11, 13). A preclinical study revealed a strong correlation between tumor angiogenesis and immune suppression (14). The aberrant tumor vasculature facilitates immune evasion by impeding the transportation of immune cells to the tumor environment (15). Antiangiogenesis therapy can normalize the irregularities of the tumor vasculature, improve oxygenation, increase the infiltration of immune cells, and transform the tumor immunosuppressive microenvironment into an immune-supportive microenvironment, which is beneficial for immunotherapy (10, 11, 16).

Multiple clinical studies have confirmed the effectiveness and safety of ICIs when applied in conjunction with angiogenesis inhibitors in various solid tumors, including lung cancer (17), hepatocellular carcinoma (18), gastric cancer (19), and cervical cancer (20). Similar results could also be seen in the patient with MSI-H/dMMR mCRC. Additionally, a retrospective study demonstrated that patients with MSI-H metastatic gastrointestinal cancer who experienced progression after ICI treatment exhibited improved clinical outcomes when ICIs were used in conjunction with angiogenesis inhibitors compared to those receiving chemotherapy alone or in combination with the targeted therapy (21). Currently, some clinical trials are evaluating the efficacy of ICIs in conjunction with anti-angiogenic and chemotherapy in MSI-H/dMMR mCRC (22, 23).

In the KEYNOTE 177 trial, it was observed that patients with RAS mutation, treated with a single-agent ICI, were associated with poorer PFS compared to patients with the RAS wild type (5). Moreover, a cohort study revealed that the ICI treatment in patients with MSI-H/dMMR mCRC who exhibited liver metastasis had a shorter PFS (24). Furthermore, the data from preclinical research demonstrated a strong association between RAS mutation, liver metastasis, and immunosuppressive environment (25, 26). Additional research is required to validate the impact of RAS mutation and liver metastasis on the efficacy of immunotherapy.

In this case report, the patient with RAS mutation and liver metastasis did not respond to camrelizumab treatment. However, the combination of camrelizumab and bevacizumab showed remarkable clinical responses in the patient. This proves that the combination therapy may overcome ICI resistance and promote the sensitivity of unresponsive tumors to ICI in MSI-H/dMMR mCRC.

In conclusion, ICI combined with an angiogenesis inhibitor exhibited a powerful and durable antitumor response in a dMMR mCRC patient after ICI therapy failure. Further investigations are required to validate the efficiency of combining ICIs and anti-angiogenesis therapy in MSI-H/dMMR mCRC patients with ICI resistance.

## Data Availability

The original contributions presented in the study are included in the article/supplementary material. Further inquiries can be directed to the corresponding author.
